# Supplementary material for: Antifungal Testing of Vaginal Candida Isolates in Pregnant Women: A Retrospective, Single-Center Study in Adana, Türkiye
Source: J Fungi (Basel). 2025 Jan 24;11(2):92. doi: 10.3390/jof11020092 (PMC11856381; doi:10.3390/jof11020092)
Supplement: Supplementary file 1 [file jof-11-00092-s001.zip › jof-3365657-supplementary.pdf]

Table S1. Threshold values for interpreting disk diffusion tests used in this study.

Antifungals	Disk concentration	Inhibition zone diameter (mm)		
		R	SDD	S
Fluconazole[a]	25 µg	≤ 14	15–18	≥ 19
Itraconazole[c]	10 µg	≤ 9	10–14	≥ 15
Ketoconazole[b]	50 µg	< 10	10–20	> 20
Myconazole[b]	50 µg	< 10	10–20	> 20
Clotrimazole[c]	50 µg	≤ 11	12–19	≥ 20
Nystatin[c]	100 U	≤ 10	11–14	≥ 15

[a]CLSI, 2009; [b]Dota et al., 2011; [c]Khan et al., 2018. R, Resistant; SDD, Susceptible-dose-dependent; S, Susceptible.

Table S2. Threshold values for interpreting broth microdilution tests in this study.

	FLU			ITR		
	S	SDD	R	S	SDD	R
C. albicans	≤2	4	≥8	≤0.12[b]		
C. glabrata		≤32	≥64			4 µg[b]
C. krusei			[a]			2 µg[b]

[a] All *Candida krusei* isolates were considered resistant to fluconazole.[b] Pfaller, 2012**.** R, Resistant; SDD, Susceptible-dose-dependent; S, Susceptible**;** FLU, Fluconazole; ITR, Itraconazole.

Table S3. Mean inhibition zone diameters of vaginal *Candida* isolates for six antifungals at pH 4 and pH 7.

	C. albicans **(n=29)**		**Petite *C. glabrata*** **(*n*=26)**		**Non-petite *C. glabrata*** **(*n*=26)**		***C. krusei*** **(*n*=13)**	
	**pH 7**	**pH 4**	**pH 7**	**pH 4**	**pH 7**	**pH 4**	**pH 7**	**pH 4**
FLU	30.5±7.1	24.2±7.8	16.7±5	15.5±5.1	17.9±9	11.1±5.1	7.4±1.2	14.1±2.7
ITR	18.9±5.1	15.5±5.2	9±2.2	8± 2.5	10±3.9	7±1.7	9.8±1.9	8.2±1.7
KTC	27.5±10.4	21.6±7.6	23.8±4.6	14.8±4.7	26.5±6	9.9±3.6	23.4±4.4	14.6±2.8
MCZ	24.2±6.7	37.2±5.6	23.3±3.6	37.3±4.1	26.3±4.4	35.8±3.7	10.7±3.6	33.8±4.1
CLT	28.5±7.1	31.5±5.7	18.8.±4.7	26.6±6.3	17.5±6.5	15.8±7	26±3.1	35.6±2.4
NY	18.8±2.4	22.6±1.6	19.4±2.1	22.7±1.5	22.0±1.8	22.6±2.1	16.6±2.1	17.7±2

[§]M±SD, The arithmetic mean and standard deviation values of the inhibition zone diameters were presented (mm). FLU, Fluconazole; ITR, Itraconazole, CLT; Clotrimazole, KTC, Ketoconazole; MCZ, Miconazole; NY, Nystatin.

Table S4. Correlation and partial correlation coefficients between the inhibition zone diameters at pH 4 and 7 for all isolates.

	Correlation Coefficient	Partial Correlation Coefficient	Correlation Coefficient			
	**All isolates**	**Controlling for microorganism**	***C. albicans***	**Petite *C. glabrata***	**Non-petite *C. glabrata***	***C. krusei***
FLU	**0.655****	**0.433****	**0.655****	**0.849****	0.156	-0.013
ITR	**0.822****	**0.735****	**0.631****	**0.950****	**0.550****	0.479
KTC	**0.401****	**0.396****	**0.587****	0.254	**0.397***	-0.090
MCZ	**0.333****	**0.267****	0.235	**0.473***	0.352	0.162
CLT	**0.574****	**0.550****	0.226	**0.485***	0.373	0.136
NY	**0.378****	**0.446****	0.149	0.026	**0.521****	-0.358

* Correlation is significant at the 0.05 level. ** Correlation is significant at the 0.01 level.

FLU, Fluconazole; ITR, Itraconazole, CLT; Clotrimazole, KTC, Ketoconazole; MCZ, Miconazole; NY, Nystatin.

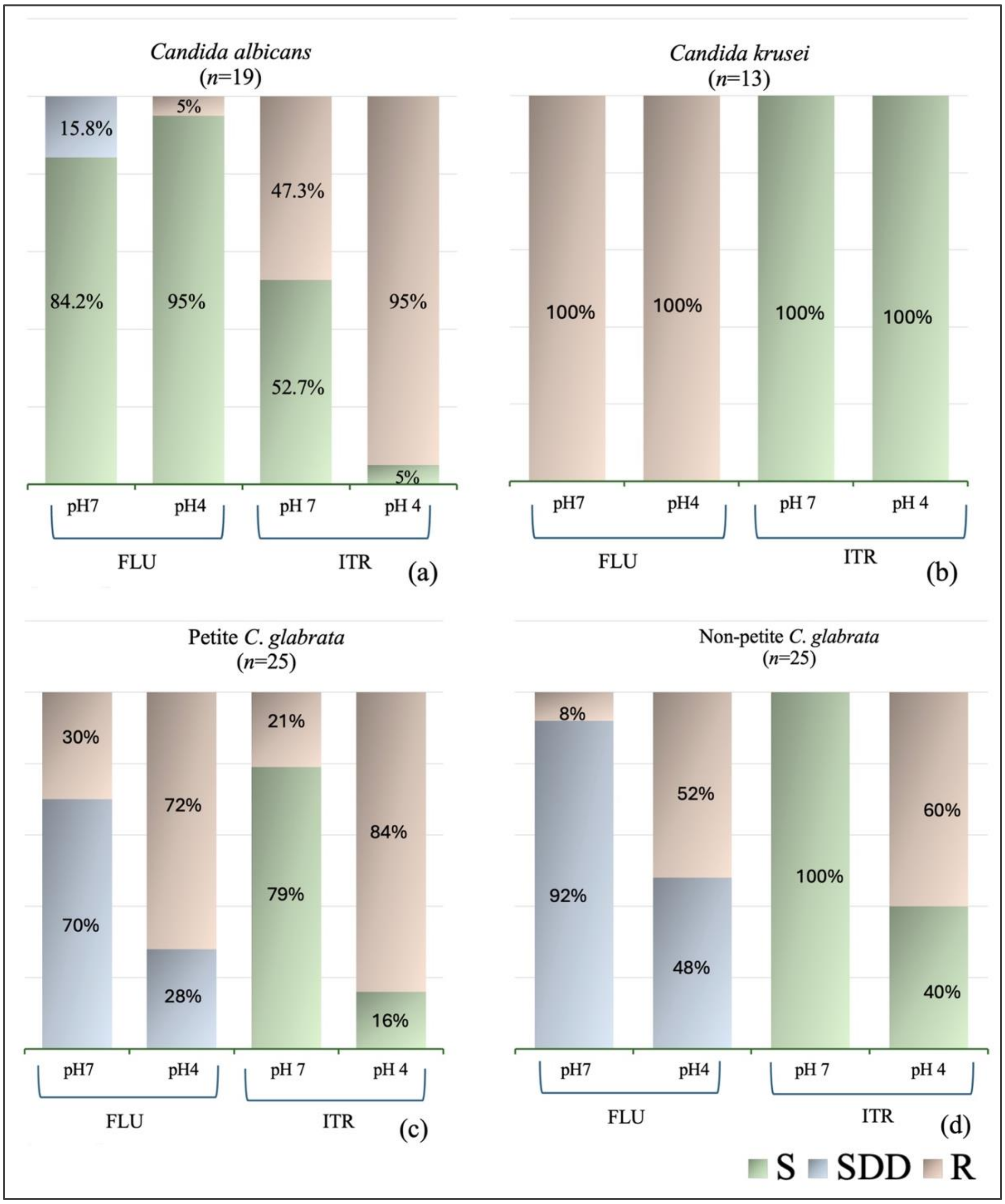

Figure S1. Broth microdilution method results for vaginal *Candida* isolates at pH 4 and pH 7: (a) *Candida albicans*, (b) *Candida krusei*, (c) petite *Candida glabrata*, (d) *non*-petite *Candida glabrata*. R, Resistant; SDD, Susceptible-dose-dependent; S, Susceptible; FLU, Fluconazole; ITR, Itraconazole.
